# Supplementary material for: Exploring the use of a digital therapeutic intervention to support the pediatric cardiac care journey: Qualitative study on clinician perspectives
Source: PLOS Digit Health. 2023 Dec 11;2(12):e0000371. doi: 10.1371/journal.pdig.0000371 (PMC10712855; doi:10.1371/journal.pdig.0000371)
Supplement: S1 Text — (DOCX) [file pdig.0000371.s001.docx]

|  |
| --- |
| Interview Guide for “MEDLY-Peds”: |

## **Clinician Interview Guide**

Section 1: Introduction

1. How many adolescent patients with heart failure are you caring for?
2. How long have you been caring for them?

Section 2: Heart Failure Management – Care Challenges and Opportunities

1. Who would you consider to be the most responsible physician for managing your patients’ heart failure? (i.e. cardiologist, family doctor, etc.)
   1. How often do your patient’s visit them/yourself?
2. What is the current model of care for HF management in adolescents (i.e. self-monitoring of nutrition, diet, weight, exercise, etc.)?
   1. With the diverse needs of the adolescent population, does their model of care change over time?
      1. Is there a current process for their transition into adult care? If so, what? If not, why not?
3. What metrics or parameters are typically reviewed to assess patient’s condition?
   1. How often are patients followed up?
   2. How long are patients followed typically?
4. What is expected of the patient in regard to their care management (e.g. self-monitoring of parameters, nutrition / diet, exercise, etc.)?
   1. Do their care needs responsibilities change over time? If so, why?
5. What is expected of the caregiver (e.g. how involved are caregivers in the adolescent’s care)?
   1. How involved do you want the caregiver to be?
   2. Does their involvement change over time? If so, why?
6. Do you think your patient, or their caregiver find any of their care tasks challenging, unnecessary or tiring?
7. Do you provide or recommend any support services to assist your patients with their care? (i.e. remote monitoring, education, help desk, etc.)
   1. If yes: What type of services? Who provides services? (doctors, nurses, social workers, etc.) What did you like/dislike about those services?
   2. If no: Why not?

Section 3: Use of Technology for Care

1. Do you find your patients generally use a mobile phone or tablet?
   1. If yes: What do they usually use it for?
   2. If no: Do you think they would feel comfortable using one if available?
2. Have your patients ever used any apps to track or manage their health? Or have you recommended any for them or their caregiver to use?
   1. If yes: What was it for and what was their experience with it (i.e. easy, understandable)?
      1. What did they like or not like about it?
      2. Do they still use it? Or if not, what made them, stop using it?
   2. If no: Do you see this as something they would be comfortable using?
3. Based on the model of care, and what is known about the existing heart failure digital therapeutic system, is there value in adapting this system to help adolescent patients with HF and their clinicians better manage their condition in the home or community?
   1. Is a remote monitoring feature (data sent to the healthcare provider), currently central to the adult HF system, needed in pediatric populations?
   2. What features or components of the adult HF TM system align with the model of care / needs of the adolescent population?
   3. Are there some confliction or diverse needs of the adolescent population that are not met by the existing HF TM system?
      1. What features would need to be added/removed/modified to make the system more appealing to them?
4. For the conflicting or diverse needs of the adolescent population, what are the product requirements for a TM system for adolescents with HF?
   1. What are the requirements for the clinician-facing technology (i.e. clinician dashboard)?
   2. What are the requirements for the patient-facing technology (i.e. smartphone application)?
   3. Is there a unique platform required for caregivers to participate?
5. Are there any reasons that you think would lead them to stop using the app?

- Is there anything else you would like to add or is there something you consider important that we have not discussed today?
