# Supplementary material for: Exploring the use of a digital therapeutic intervention to support the pediatric cardiac care journey: Qualitative study on clinician perspectives
Source: PLOS Digit Health. 2023 Dec 11;2(12):e0000371. doi: 10.1371/journal.pdig.0000371 (PMC10712855; doi:10.1371/journal.pdig.0000371)
Supplement: Attachment — Submitted filename: PloS Digital Health Response to Reviewers.docx [file pdig.0000371.s002.docx]

Dear Reviewers:

Please find responses regarding our manuscript, “Exploring the Use of a Digital Therapeutic Intervention to Support the Pediatric Cardiac Care Journey: Qualitative Study on Clinician Perspectives" submitted for publication as an Original Article in PloS Digital Health.

1. “Throughout the paper, the authors refer to their institutionalized program known as Medley. Sometimes when they refer to it, they italicize it, and other times they do not. The authors should be consistent throughout the paper”

**Response: We have italicized the program name throughout the paper for consistency**

2. “The first section in the method section discusses the setting/intervention. At the end of this section, the authors discuss the study aim specifically related to how they will assess their Medley program. These paragraphs would be better suited in the introduction section. However, the introduction section was already at an appropriate length, so I encourage the authors to streamline it.”

**Response: We have revised the end of this section to streamline the description provided. Similar to other mHealth or digital health focused studies, we have constructed our manuscript to describe the setting/intervention in the methods section as it provides the contextual details pertaining to the overarching study aim.**

3. “In the recruitment of participant section, line 142, the authors report congenital heart disease requiring frequent hospital visits. It would be helpful to know what the authors defined as frequent.”

**Response: We have added a description to indicate frequent hospital visits as greater than 3-4 visits/year for CHD patients. However, in reality the indication of more frequent visits may be subjective to the care provider and the complexity of the patient.**

4. “The authors provided a very detailed data collection and analysis which I found extremely interesting and could be expanded. Would it be possible for the authors to include the interview questions? It would also be interesting to include the response scoring and show how many times the five themes that were reported were identified, etc.”

**Response: We have added the interview guide as a supplementary material to the manuscript. Given that the process of data analysis involved combining feedback from the clinicians across different themes, we believe a scoring of the reported themes would not be of value and may potentially bias the strength of each theme within the narrative of the overall study findings.**

5. “Table 1 does not add much. If the authors could expand more on how many years of practice each clinician had or some other demographic details, it may make the table more interesting.”

**Response: Given the limited size of the Heart Function program, we have limited the demographic details within the table to prevent any identifiable data from being connected to the clinicians. Instead, we have removed the table as the first paragraph of the results paper describes the expertise of the participants, and the table does not provide any additional context.**

6. “It would also be helpful to know why the authors were only able to get eight people to consent for the study.”

**Response: Our team aimed to recruit between 7-10 clinicians of varying clinical expertise (cardiologist, nurse practitioner, fellow) for this study. The study team began to recognize repeated feedback after 6 interviews, to ensure data saturation an additional two clinicians were interviewed, whereby the team concluded no new perspectives or context was provided.**

7. In the results section, I fear that the direct quotes, change the tone of the paper from more academic to read more like a news article or journalism article as opposed to an academic scientific paper. I am not suggesting removing them completely, but perhaps paraphrasing them to support what the authors' point is. This also made the results section extremely lengthy, and I think many readers would skip down to the discussion section.

**Response: We have shortened some of the quotes to reduce the length of the manuscript. Within qualitative papers, direct quotes are often used to help strengthen the contextual significance of the participant’s feedback. We have paraphrased the majority of content of the included quotes within the paragraph above the quote section.**

8. The Introduction focuses largely on congenital heart disease even though more than half of heart failure in adolescents is secondary to acquired heart disease. The third sentence of the first paragraph of the Introduction is very confusing as it suggests that patients with CHD are at risk for heart failure due to cardiomyopathy, myocarditis and other inflammatory or infectious etiologies as these are typically causes of acquired heart disease leading to heart failure. The Authors need to make a very clear distinction in the Introduction between heart failure due to acquired heart disease and heart failure due to congenital heart disease. Additionally, the Authors should confirm that the statistics noted are representative of pediatric heart failure in general and not just secondary to CHD.

**Response: We have revised the introduction section to differentiate the causes of heart failure and have changed the language to note that the statistic are representative of pediatric heart failure.**

9. One other area for exploration in the consideration of adoption of digital health technology in adolescents with heart failure secondary to congenital heart disease relates to the potential for neurocognitive deficits. These neurocognitive deficits may be associated with a history of palliated cyanotic heart disease (e.g., HLHS) or in patients with trisomy 21, which studies have demonstrated a high rate of heart failure in adult survivors with CHD. This is hinted at in the discussion related to school performance (ii. Patient care needs change over time) but a more nuanced discussion may be helpful.

**Response: We have added a section describing the importance of exploring the impact of neurocognitive deficits within the discussion section.**

10. “Abstract, Results – It is stated that “four themes were identified” though five themes are listed. Within the body of the manuscript, it is correctly stated to be “five themes”.”

**Response: We have corrected this within the abstract to state five themes.**

11. “Results, Multidisciplinary model of care section, paragraph 1, sentence 2 – “patient” should be “patients”.”

**Response: We have corrected this section to state ‘patients’.**

Thank you,

Sahr Wali, PhD

Scientific Associate II

Ted Rogers Centre for Heart Research

University Health Network
